# Association between Epstein-Barr virus and Thymic epithelial tumors: a systematic review

**DOI:** 10.1186/s13027-019-0254-5

**Published:** 2019-11-06

**Authors:** Guofei Zhang, Zipu Yu, Gang Shen, Ying Chai, Chengxiao Liang

**Affiliations:** 1 Department of Thoracic Surgery, the Second Affiliated Hospital, College of Medicine, Zhejiang University, # 88 Jiefang Road, Hangzhou, 310009 China; 2 Department of Surgery, Zhejiang Hospital, # 12 Lingyin Road, Hangzhou, 310013 China

**Keywords:** Epstein-Barr virus, Thymic epithelial tumor, Thymic carcinomas, Myasthenia gravis, Systematic review

## Abstract

The possible role of Epstein-Barr virus (EBV) in the pathogenesis of thymic epithelial tumors (TET) remains controversial. This study aimed to determine the prevalence of EBV in TET. We conducted a systematic review of relevant English-language studies published between January 1980 and December 2013. Effect size was calculated as event rates (95% confidence interval [CI]) by homogeneity testing using Cochran’s Q and I^2^ statistics for benign TET, benign TET with myasthenia gravis (MG), and thymic carcinoma (TC). Among 136 potentially relevant studies, 22 met the inclusion criteria. Despite a considerable degree of heterogeneity, the pooled estimated incidences were 9% (95% CI, 1–23%), 20% (95% CI, 0–54%), and 6% (95% CI, 0–21%) for benign TET, benign TET with MG, and TC, respectively. There was significant heterogeneity among studies that used in situ hybridization (ISH) for both benign TET and benign TET with MG. According to the random-effects model, studies employing ISH yielded lower point estimates of EBV prevalence (5%) than those employing other methods (33%). Using the random-effects model, we found a lack of significant heterogeneity among studies from different geographic regions (*p* = 0.0848). Further, 12 of 23 lymphoepithelioma-like carcinoma (LELC) cases tested EBV-positive. The prevalence of EBV in benign TET with or without MG was lower than in nasopharyngeal carcinoma, suggesting that EBV plays a minor role in TET pathogenesis. Although the prevalence of EBV in TC was also low, EBV may play an important causal role in LELC. Further research is needed to clarify these associations.

## Introduction

Thymic epithelial tumors (TET) are the most common type of primary tumor in the anterior mediastinum, although they are rare. They can be benign (e.g., thymomas, thymic cysts, thymic hyperplasia, and thymolipomas) or malignant (e.g., thymic carcinomas [TCs] and thymic carcinoids). Moreover, approximately 30 to 50% of patients with thymomas have myasthenia gravis (MG), and approximately 80% patients with MG have thymic abnormalities, including thymoma and hyperplasia [1]. Although the mechanism underlying TET is not known, genetic and environmental factors are both suspected to be involved in the etiology of TET [2, 3]. Additionally, infection by common viruses is considered to be a possible environmental factor leading to autoimmunity via alteration of the host immune system.

The Epstein-Barr virus (EBV) is well known for its association with several epithelial neoplasms, inclusive of nasopharyngeal carcinoma, gastric carcinoma, and lymphoepithelioma-like carcinoma (LELC) of several sites (e.g., stomach, salivary gland, and lung) [4]. Next to the direct involvement in the pathogenesis of infectious mononucleosis, EBV is associated with a wide variety of neoplastic lymphoproliferative disorders, including Burkitt lymphoma, Hodgkin disease, post-transplantation lymphoproliferative disease, and non-Hodgkin’s lymphoma arising in immunocompromised patients, as shown in seroepidemiological, immunological, and DNA studies [5].

Recently, discussion of the possible role of EBV in TET pathogenesis has been stimulated by a study reporting EBER-positive cells in all 17 MG thymic specimens analyzed [6]. Many thymic cells showed positive staining for the EBV-encoded nuclear antigen and latent membrane proteins 1 and 2A. A follow-up study suggested that EBV infection may be involved in the onset or perpetuation of MG [7]. These results, however, could not be reproduced by other groups and thus remain controversial. Two subsequent independent studies did not find evidence of EBV infection in thymic specimens of patients with MG [8, 9].

Since then, several new reports have been published, but a consistent conclusion has not yet been reached. To fill this gap, we conducted a quantitative systematic review of the accumulated evidence on EBV in TET in the published literature, without any restrictions concerning the EBV detection method or geographic origin of the study.

## Materials and methods

### Literature search and selection criteria

This systematic review was conducted according to the Meta-analysis of Observational Studies in Epidemiology guidelines [10]. A comprehensive search of PubMed, Embase, Ovid, and Google Scholar was performed to identify studies that assessed the presence of EBV infection in human subjects with TET with or without MG published between January 1, 1980 and September 31, 2019. The reference lists of relevant papers and reviews were examined to identify further articles. The key search terms used were Epstein-Barr virus, EBV, thymic, thymus, myasthenia gravis, MG, and thymomas.

Only papers published in English were considered. The included studies provided the exact numbers of analyzed cases and cases testing EBV-positive, which were necessary for calculation of the event rates (EBV prevalence) and their 95% confidence intervals (95% CI). In addition, the included studies provided a clear description of the EBV detection methods and a definite histopathological diagnosis of the thymus. However, studies performed on human cell lines and those with an unknown pathology of the thymus were excluded from the systematic review. Studies with a sample size < 5 cases were also excluded except for those evaluating patients with LELC because of its rarity. Additionally, studies that used enzyme-linked immunoassays for EBV detection and those that evaluated blood samples without detection in thymic tissue were also excluded from the systematic review.

### Data extraction

The studies were evaluated critically by two authors (ZG and LC). If disagreement persisted after the complete manuscript was studied, a third reviewer was consulted. From the summary and/or body of the text of each eligible study, we obtained the following information: author of the publication, geographic region of the study, type of specimen, histological type of thymoma analyzed, EBV detection method, total number of cases analyzed, number of cases testing EBV-positive, and percent EBV positivity.

### Statistical analysis

The analysis was conducted using R version 3.6.1 for Windows. No prospective studies that reported EBV status in both TET cases and controls were available. Therefore, odds ratios could not be calculated in an appropriate manner. The proportion of EBV-positive cases was analyzed in 3 groups: benign TET, benign TET with MG, and TC.

We estimated the prevalence using the double arcsine transformation because the inverse variance weight in fixed effects (FE) is suboptimum when dealing with binary data with low prevalence. Additionally, the transformed prevalence was weighted very slightly towards 50%, and studies with a prevalence of zero could thus be included in the analysis [11, 12]. The Q statistics were calculated to test for heterogeneity among the studies included in this analysis and were considered statistically significant when *p* < 0.05. The I^2^ statistic was also used to assess the extent of between-study heterogeneity. The DerSimonian and Laird (random-effects) method was selected when there was evidence of statistical heterogeneity. The robustness of the pooled proportions was explored through sensitivity analyses. Sources of heterogeneity were explored using subgroup and meta-regression analyses. Specifically, we chose to study factors related to the EBV detection method and geographic origin.

## Results

### Search results

Our search, which was performed on October 2, 2019, identified 136 articles. Of these articles, 115 were excluded on the basis of their titles or abstracts, and 21 were selected for full-text review. A manual review of the references of relevant publications was also performed to identify additional studies, which yielded 2 additional articles. Ultimately, 22 studies fulfilled the inclusion and exclusion criteria and were included in this review; 15 were larger series related to the prevalence of EBV in thymic tissues, and 7 were case reports on EBV in LELC. Table 1 summarizes the detection of EBV in TET using different detection techniques.

**Table 1:** Studies reporting EBV detection in TET

Year	Study (reference)	Country	Detection method	Pathological type	MG	No. assessed	No. positive
1988	McGuire et al. [13]	China	SBH	T, LT, TI	MG	11	6
1990	Inghirami et al. [14]	USA, Italy	SBH, PCR	T	MG	16	0
1990	Borisch et al. [15]	Germany	ISH, SBH	NT, TH	MG	51	0
1992	Mann et al. [16]	USA	ISH	LT, T/TC	–	40/7	0/1
1993	Wu et al. [17]	China	ISH	T/TC	–	21/20	0/1
1993	Fujii et al. [18]	Japan	ISH	T/TC	–	7/8	0/1
2000	Hishima et al. [19]	Japan	ISH	T/TC	–	18/9	0
2000	Engel et al. [20]	Denmark	ISH	T/TC	–	105/52	0
2001	Tateyama et al. [21]	Japan	ISH	T/TC	–	9/2	4/1
2002	Chen et al. [22]	China	ISH, PCR	T/TC	–	78/21	0/6
2010	Cavalcante et al. [6]	Italy	ISH, IH, PCR	TH, LT, TI	MG	17	17
2011	Meyer et al. [8]	Germany	ISH, IH	LT	MG	44	0
2011	Cavalcante et al. [7]	Italy	PCR	TH, LT, TI	MG	19	12
2011	Kakalacheva et al. [9]	Europe	ISH, IH, PCR	LT	MG	16	1
2013	Jing et al. [23]	China	ISH, IH	TH	MG	30	0
2017	Cavalcante et al. [23]	Italy	PCR				

EBV, Epstein-Barr virus; TET, thymic epithelial tumors; SBH, Southern blot hybridization; PCR, polymerase chain reaction; ISH, in situ hybridization; IH, immunohistochemistry; MG, myasthenia gravis; LT, lymphofollicular thymitis; T, thymoma; TH, thymic hyperplasia; TI, thymic involution; NT, normal thymus; TC, thymic carcinoma

### Prevalence of EBV in benign TET

**Fig. 1:**
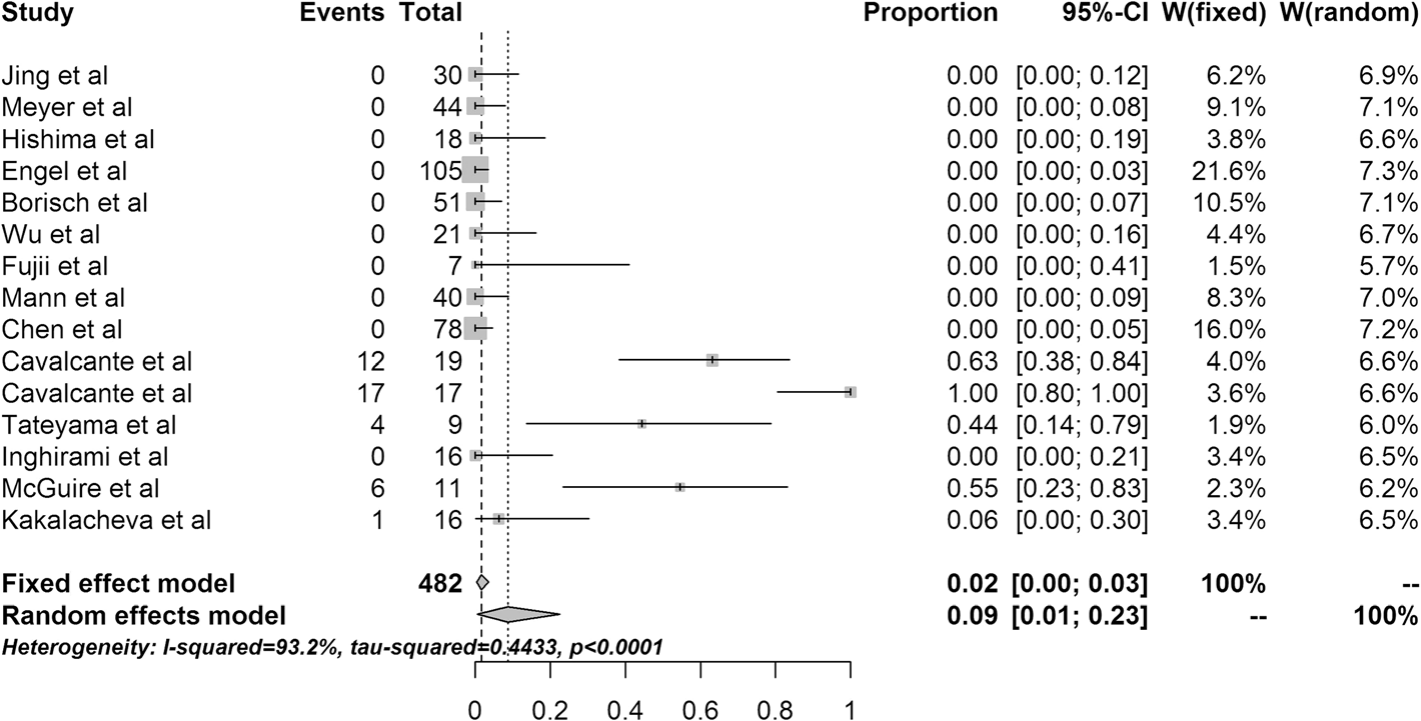
Forest plot of the 15 studies reporting Epstein-Barr virus in benign thymic epithelium tumors. CI, confidence interval

To date, 15 published studies have analyzed the evidence for EBV involvement in benign TET. The reported incidence of EBV in benign TET varied markedly across individual studies; 10 studies reported a prevalence of 0% and one study reported a prevalence of 100%. As significant heterogeneity was observed across the included studies, measured by Cochran’s Q statistics with *p* < 0.0001, we used a random-effects (RE) model to pool the data. Therefore, the crude EBV positivity (40/482) translated to an EBV prevalence of 0.09 (95% CI 0.01–0.23) using the RE model (Fig. 1).

We hypothesized that appropriate selection of the detection method is important for accurate estimation; in situ hybridization (ISH) is the gold standard for EBV detection, most of the studies used ISH. However, significant heterogeneity was still observed among studies using ISH (*p* < 0.001, Table 2). ISH gave lower point estimates of EBV prevalence (5%) than other methods (33%) when using the RE model. When the data were analyzed according to regional group, the between-strata comparison using the RE model was not significant at *p* = 0.0848, indicating that studies from different geographic regions were not significantly different. The incidence was 5% (95% CI, 0–20%) for Asian cases, 19% (95% CI, 0–56%) for European cases, and 0% (95% CI, 0–3%) for other cases (Table 3).

Although there was some evidence of publication bias (Egger’s *p* = 0.023) there was no evidence for publication bias among studies (*n* = 12) that used the ISH (Egger’s *p* = 0.078). Sensitivity analyses were conducted to explore the robustness of these observations, and the results seemed robust to all one-by-one removals of studies, with no change in the magnitude and precision of the FE and RE summary point estimates of the effect size.

### Prevalence of EBV in MG

**Fig. 2:**
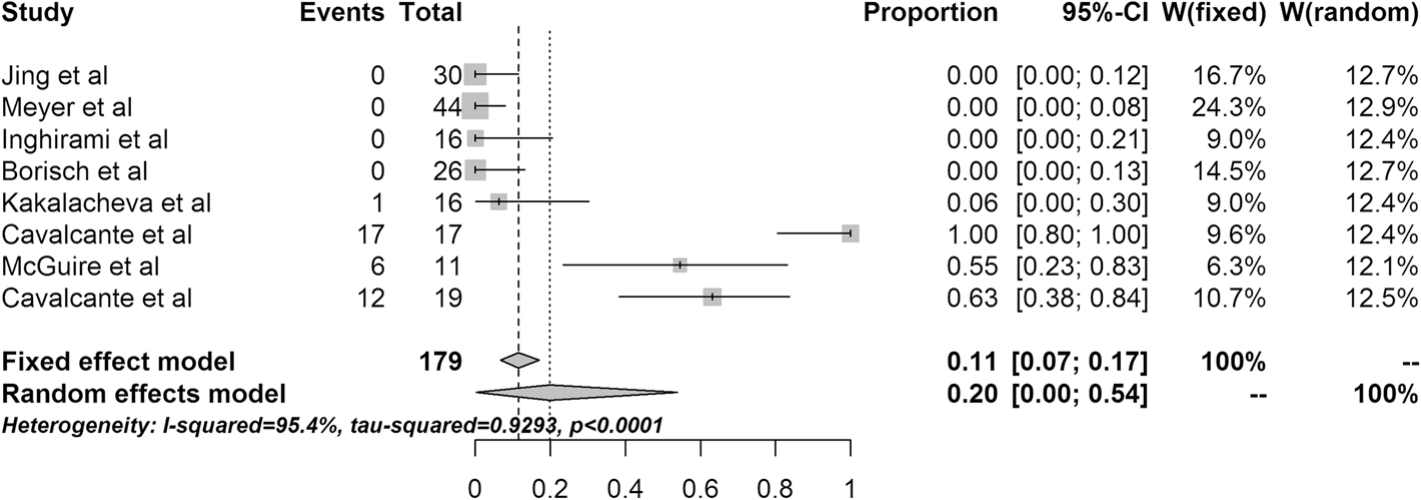
Forest plot of the 8 studies reporting Epstein-Barr in myasthenia gravis. CI, confidence interval

In total, 8 studies analyzed the association between EBV and MG, irrespective of the pathology of TET. Significant heterogeneity was observed among the studies, as measured by Cochran’s Q statistics, with *p* < 0.001. The crude EBV positivity (36/179) translated to an EBV prevalence of 0.20 (95% CI 0–54%) using the RE model (Fig. 2). Among the 8 studies, the incidence of EBV in MG varied. That is, 4 studies reported no association between EBV and MG, but one study in Caucasian patients reported 100% positivity for EBV.

When the studies were stratified according to EBV detection technique, the between-strata comparison using the RE model was not significant at *p* = 0.5233, indicating that there was no difference among studies using different EBV detection techniques (Table 4). However, the detection rates tended to be lower in studies that used ISH, although even then a wide variation in detection rate (from 0 to 100%) was evident. The number of studies was too low to draw definite conclusions on the role of EBV in the etiology of MG when the studies were stratified by geographic origin.

### Prevalence of EBV in TC

**Fig. 3:**
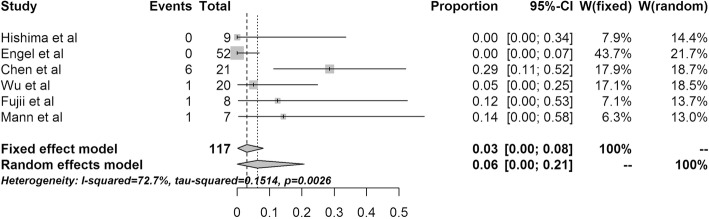
Forest plot of the 6 studies reporting Epstein-Barr in thymic carcinoma. CI, confidence interval

Six studies reported an association between EBV and TC, and all of them used ISH. Significant heterogeneity was observed between the studies, as measured by Cochran’s Q statistics, with *p* = 0.0026. The crude EBV positivity (9/117) translated to an EBV prevalence of 0.06 (95% CI 0–0.21) using the RE model (Fig. 3). Of the 6 studies, 2 reported no association between EBV and TC, and 3 reported only 1 positive case.

LELC is an undifferentiated or poorly differentiated squamous cell carcinoma associated with a prominent component of reactive lymphocytes and plasma cells. In the English literature, we found a total of 23 cases in which the association between EBV and LELC was investigated; 12 of these cases were positive for EBV, including 8 men and 4 women with a mean (± standard deviation) age of 26.75 ± 19.4 years (range: 10 to 73 years) (Table 5). Therefore, although the EBV positivity was very low in TC, EBV likely contributes to the development of thymic LELC.

**Table 2:** Analysis of the 15 studies stratified according to EBV detection method

Detection method	No. of studies	Events	Sample size	Point estimates of event rates (FE)		Point estimates of event rates (RE)		Homogeneity (Cochran’s Q)	*I* ^2 1^	Homogeneity (*p*-value)
				Point estimate	95% CI	Point estimate	95% CI			
ISH	12	22	436	0	0–0.02	0.05	0–0.17	144.03	92.4	< 0.0001
Other	3	18	46	0.32	0.19–0.47	0.33	0–0.84	24.96	92	< 0.0001
Summary	15	40	472	0.02	0.01–0.03	0.08	0.01–0.21	206.73	93.2	< 0.0001
Total within (FE)								168.99		< 0.0001
Total between (FE)								37.74		< 0.0001
Total between (RE)								1.69		0.1931

FE, fixed-effects model; RE, random-effects model. EBV, human papillomavirus; ISH, in situ hybridization

1 Only calculated for the fixed-effects model

**Table 3:** Analysis of the 15 studies stratified by geographic origin

Geographic origin	No of studies	Events	Sample size	Point estimates of event rates (FE)		Point estimates of event rates (RE)		Homogeneity (Cochran’s Q)	*I* ^2 1^	Homogeneity (*p*-value)
				Point estimate	95% CI	Point estimate	95% CI			
Asia	7	10	174	0	0–0.03	0.05	0–0.2	37.38	83.9	< 0.0001
Europe	6	30	252	0.04	0.02–0.07	0.19	0–0.56	164.51	97	< 0.0001
Other	2	0	56	0	0–0.03	0	0–0.03	0.09	0	0.7628
Summary	15	40	472	0.02	0–0.03	0.09	0–0.23	206.73	93.2	< 0.0001
Total within (FE)								201.98		< 0.0001
Total between (FE)								4.75		0.093
Total between (RE)								4.93		0.0848

FE, fixed-effects model; RE, random-effects model

1 Only calculated for the fixed-effects model

**Table 4:** Analysis of the 8 studies stratified by the EBV detection method

Detection method	No. of studies	Events	Sample size	Point estimates of event rates (FE)		Point estimates of event rates (RE)		Homogeneity (Cochran’s Q)	*I* ^2 1^	Homogeneity (p-value)
				Point estimate	95% CI	Point estimate	95% CI			
ISH	5	18	133	0.06	0.02–0.12	0.14	0–0.57	111.55	96.4	< 0.0001
Other	3	18	46	0.32	0.19–0.47	0.33	0–0.84	10.34	92	< 0.0001
Summary	8	36	179	0.11	0.07–0.17	0.20	0–0.54	152.8	95.4	< 0.0001
Total within (FE)								136.51		< 0.0001
Total between (FE)								15.29		< 0.0001
Total between (RE)								0.41		0.5233

FE, fixed-effects model; RE, random-effects model. EBV, Epstein-Barr virus; ISH, in situ hybridization

1 Only calculated for the fixed-effects model

**Table 5:** Relationship between EBV and LELC reported in the literature

Year	Technique	Cases	EBV-positive cases	Age/sex	Reference
1985	SB	1	1	19/M	Leyvraz et al. [24]
1988	SB	1	1	30/F	Dimery et al. [25]
1988	SB	1	1	73/F	McGuire et al. [13]
1992	PCR	4	1	10/M	Matsuno et al. [26]
1992	ISH	4	1	26/F	Mann et al. [16]
1993	SB	1	1	15/M	Patton et al. [27]
1993	ISH	5	1	19/M	Wu et al. [17]
1993	ISH	1	1	13/F	Fujii et al. [18]
1996	ISH	1	1	14/M	Niehues et al. [28]
2001	ISH	2	1	59/M	Tateyama et al. [21]
2009	IH	1	1	26/M	Koppula et al. [29]
2012	PCR	1	1	17/M	Januszkiewicz et al. [30]
Total	–	23	12	–	–

EBV, Epstein-Barr virus; LELC, lymphoepithelioma-like carcinoma

## Discussion

The role of EBV in the etiology of TET has attracted increasing interest since the publication of several contradictory reports on the incidence of EBV in TET [6–9]. However, until now, no systematic review has been published. The present study was a systematic review of published studies on the role of EBV in the etiology of TET. The studies analyzed benign TET, benign TET with MG, and TC. Moreover, the study-level covariates considered in this meta-analysis were the EBV detection method and the geographic origin of the study. Although there was a considerable degree of heterogeneity, the pooled estimated incidences of all 3 categories were 9% (95% CI, 1–23%), 20% (95% CI, 0–54%), and 6% (95% CI, 0–21%) in benign TET, benign TET with MG, and TC, respectively. In addition, studies that evaluated the association between EBV and LELC were also analyzed: 12 of the 23 LELC cases were EBV-positive.

Heterogeneity across study estimates was an important factor limiting the interpretation of our results. Although we attempted to minimize heterogeneity in this review by grouping studies according to the EBV detection method, marked heterogeneity existed in studies of benign TET with and without MG, as assessed by the Q test and I^2^ index. Although ISH detection of EBERs is a sensitive and specific method for in situ detection of EBV infection in routinely processed tissues and ISH-based studies comprised the bulk of the studies in the present review (12/15 studies), the RE model suggests a lack of true heterogeneity between the studies using different EBV detection techniques, as indicated by the non-significant *p*-value for homogeneity (*p* = 0.1931) in the between-study summary comparison.

An alternative view suggests that the variability in EBV prevalence may be related to the different geographic regions of the studies. To test this hypothesis, we performed our analyses stratified according to the geographic origin of the studies. However, when the RE model was applied to calculate the summary statistics, a non-significant (*p* = 0.0848) summary homogeneity *p*-value was obtained for the between-strata comparison, which suggests that the variation in EBV prevalence between different geographic regions is not statistically significant. It should also be emphasized, however, that relatively few studies have been published from other geographic regions.

Some evidence for publication bias was detected, but publication bias usually had an insignificant effect on the adjusted point estimates in the stratified meta-analysis. Importantly, there was no evidence of publication bias among the ISH-based studies. As the number of studies was too low for analysis when the studies were stratified by geographic origin, we could not evaluate potential publication bias among studies of different geographic origins. In the sensitivity analysis, all of the meta-analytic results seemed robust to all one-by-one removals of studies, with no change in the magnitude and precision of the FE and RE summary point estimates of the effect size. This finding suggests that none of the 15 studies was sufficiently influential to affect the summary point estimates of this meta-analysis.

### Strengths and weaknesses of this review

By synthesizing all of the available published data on thymomas, we have provided a more precise estimate of the prevalence of EBV in TET than was previously available. The influence of variation in study design was diluted by pooling all of the available data, and we were able to explore this heterogeneity by examining study characteristics and conducting subgroup analyses. However, our study also had some limitations. First, some of the studies were not originally designed to determine the prevalence of EBV in TET, and the unknown pathology of TET resulted in incomplete case assessment. Second, the number of studies was low; when the studies were stratified by geographic origin, the number of studies was too low to draw definite conclusions on the role of EBV in the etiology of MG. Furthermore, the published studies only represent certain geographic regions and thus did not provide information on the global status of EBV in TET. Finally, similar to most meta-analytic reviews, we pooled studies that exhibited significant heterogeneity (i.e. in the finding of EBER in TET). In order to reach conclusive evidence on this topic, it may be helpful if international experts in the field of EBV and also of TET could review the published samples.

## Conclusion

We have provided evidence regarding the prevalence of EBV in TET. Compared to the necessary role of EBV in nasopharyngeal carcinoma, which has been well documented in the literature, the prevalence of EBV in benign TET with or without MG was low, which suggests that EBV plays a minor role in the pathogenesis of TET. In the analyses stratified according to EBV detection method and geographic origin of the study, the variability among studies did not reach statistical significance when RE models were used. However, more data are necessary to better evaluate the impact of EBV in the pathogenesis of TET. Although the prevalence of EBV in TC is also low, EBV may play an important causal role in LELC. Further research is necessary to shed light on these associations.

## Data Availability

Not applicable.
